# Identification of a putative molecular regulator of cork cambium

**DOI:** 10.1186/1753-6561-5-S7-P70

**Published:** 2011-09-13

**Authors:** Andreia Miguel, Pinto C Ricardo, Brian Jones, Célia Miguel

**Affiliations:** 1 Instituto de Tecnologia Química e Biológica (ITQB)-UNL; Instituto de Biologia Experimental e Tecnológica (IBET), Av. da República, EAN, 2780-157 Oeiras, Portugal; 2 A20 John Woolley Building, 2006 University of Sydney – Australia / UPSC, Sweden

## Background

Cork oak (*Quercus suber*) is a long-living species of the Fagaceae family that highly contributes for the economy of several countries such as Portugal. It is the only plant species with a phellogen capable of a sustainable production of cork with properties suited for industry applications. Phellogen, or cork cambium, usually initiates in the subepidermis and differentiates during the first year of growth [1]. Its meristematic activity gives rise to phellem (cork) cells to the outside and to phelloderm to the inside.

It is likely that genes involved in the regulation of other plant meristems such as shoot apical meristem and vascular cambium are also involved in the regulation of phellogen. Based on its role in other meristems, and on specific expression patterns in poplar stems, we hypothesize that *SHORT-ROOT* (*SHR*), a transcription factor from the GRAS family, may be involved in the regulation of phellogen. This gene has been well described in the *Arabidopsis* root where it plays a key role in the radial patterning and in regulating the specification of the root stem cell niche [2-4]. Recently, its involvement in the root vascular system to control patterning processes [5] and in the control of proliferative cell division in developing leaves [6]has also been reported. In this work we report the cloning of *SHR* from cork oak transcriptome and the characterization of *SHR* expression patterns in the stem with the aim of investigating its putative function in the phellogen.

## Materials and methods

Through a search in public databases we have retrieved putative orthologs of *ArabidopsisSHR* gene from species with a sequenced genome including *Populus trichocarpa*, *Vitis vinifera* and *Medicago truncatula*. Based on multiple alignments at the cDNAand deduced aminoacid sequence levels, we have designed degenerate primers in order to clone and characterize *SHR* from cork oak transcriptome. The amplified PCRfragment was inserted into pCR2.1 (Invitrogen) and sequenced to determine the percent identity to known *SHR* gene sequences.

To analyze *SHR* expression patterns poplar is being used as a model tree species. Transgenic poplar plants carrying the *SHR* promoter fused to the *GUS* reporter gene have been previously generated. Stem sections collected from 1-2 year-old transgenic plants have been subject to the histochemical GUS assay and embedded in Technovit 7100 resin for detailed analysis of gene expression localization under the microscope.

Characterization of *SHR* transcript abundance in cork oak developing stems taking into account different developmental stages and collection seasons has also been performed.Transcript abundance was analysed in stem tissues from the first to the third year of growth collected between January and October from a cork oak tree in order to cover periods of active growth and dormancy. Reproduction cork was collected in July which is the period during which the debarking process is performed from cork producer trees. RNA was extracted according to a protocol described by Reid *et al.* (2006) and cDNA was successfully synthesized using the Transcriptor High Fidelity cDNA Synthesis Kit (Roche). Quantitative RT-PCRwas performed with LightCycler LC480 (Roche).

## Results

While *Arabidopsis* has one *SHR* gene sequence in its genome, at least two additional *SHR*-like sequences are present in the poplar genome. Also, more than one *SHR*-like sequence seems to be present in the cork oak transcriptome and, at the moment, one of those sequences, *QsSHR1*, consisting of 1455bp and showing strong homology to the *Arabidopsis SHR* gene, was identified, and cloned. This putative *SHR*-like gene from cork oak was found to be expressed in stems at different developmental stages as well as in reproduction cork. Furthermore, evidence of specific expression of one of the poplar *SHR*-like sequences has been found in the phellogen and lenticels of stems from transgenic poplar carrying a promoter fusion to the *GUS* reporter gene.

## Conclusions

*SHR*-like genes may be involved in even more developmental processes than previously thought. It seems plausible that in woody species such as poplar and cork oak, additional copies of *SHR* may have evolved to acquire new functions. We expect that these results, as well as results from further ongoing studies, will contribute to elucidate the putative involvement of *SHR* in the regulation of phellogen. Additionally, these studies may provide tools to be used in future strategies aiming at the improvement of cork production from cork oak.
